# Polymorphisms Related to Iron Homeostasis Associate with Liver Disease in Chronic Hepatitis C

**DOI:** 10.3390/v15081710

**Published:** 2023-08-09

**Authors:** Anna Wróblewska, Anna Woziwodzka, Magda Rybicka, Krzysztof P. Bielawski, Katarzyna Sikorska

**Affiliations:** 1 Laboratory of Photobiology and Molecular Diagnostics, Intercollegiate Faculty of Biotechnology University of Gdansk and Medical University of Gdansk, 80-307 Gdansk, Poland; anna.wroblewska@ug.edu.pl (A.W.); anna.woziwodzka@ug.edu.pl (A.W.); magda.rybicka@ug.edu.pl (M.R.); krzysztof.bielawski@ug.edu.pl (K.P.B.); 2 Division of Tropical Medicine and Epidemiology, Faculty of Health Sciences, Institute of Maritime and Tropical Medicine, Medical University of Gdansk, 81-519 Gdynia, Poland

**Keywords:** hepatitis C virus infection, single nucleotide polymorphisms, iron homeostasis, hepatocellular carcinoma

## Abstract

Dysregulation of iron metabolism in chronic hepatitis C (CHC) is a significant risk factor for hepatic cirrhosis and cancer. We studied if known genetic variants related to iron homeostasis associate with liver disease progression in CHC. Retrospective analysis included 249 CHC patients qualified for antiviral therapy between 2004 and 2014. For all patients, nine SNPs within *HFE*, *TFR2*, *HDAC2*, *HDAC3*, *HDAC5*, *TMPRSS6*, and *CYBRD1* genes were genotyped. Expression of selected iron–related genes, was determined with qRT-PCR in 124 liver biopsies, and mRNA expression of co-inhibitory receptors (*PD-1*, *Tim3*, *CTLA4*) was measured in 79 liver samples. *CYBRD1* rs884409, *HDAC5* rs368328, *TFR2* rs7385804, and *TMPRSS6* rs855791 associated with histopathological changes in liver tissue at baseline. The combination of minor allele in *HDAC3* rs976552 and *CYBRD1* rs884409 linked with higher prevalence of hepatocellular carcinoma (HCC) during follow up (OR 8.1 CI 2.2–29.2; *p* = 0.001). Minor allele in *HDAC3* rs976552 associated with lower hepatic expression of *CTLA4*. Tested polymorphisms related to iron homeostasis associate with histopathological changes in the liver. The presence of both *HDAC3* rs976552 G and *CYBRD1* rs884409 G alleles correlates with HCC occurrence, especially in the group of patients with elevated AST (>129 IU/L). rs976552 in *HDAC3* could impact immunological processes associated with carcinogenesis in CHC.

## 1. Introduction

Infection with the hepatitis C virus remains one of the main causes of chronic liver disease globally with an estimated 1.5 million new infections and 300,000 deaths from HCV-related complications occurring every year [1]. Over the last few decades tremendous advancements have been made in the diagnosis, therapy, and prevention of HCV infection. Still, however, further improvements in our understanding of disease pathophysiology are necessary to explain differences in disease trajectory and highly variable clinical outcome [2]. The long-term effects of chronic HCV infection (CHC) in the liver range from minimal necro-inflammatory changes to advanced fibrosis, cirrhosis, and hepatocellular carcinoma (HCC). HCC is the most common type of hepatic cancer and is responsible for 75–85% of all liver cancer cases worldwide [3]. HCV infection is one of the leading etiologic agents for HCC and its risk in CHC increases as liver fibrosis progresses, reaching 1–4% annual HCC incidence in cirrhotic patients [4]. Liver disease progression and extrahepatic complications of the infection, such as lymphoproliferative diseases, can occur in CHC patients regardless of therapeutically induced sustained viral response (SVR).

Regulation of normal body iron metabolism occurs through hepcidin hormone produced mainly in the liver. Hepcidin interacts with ferroportin, the major cellular transmembrane iron exporter, leading to its internalization and degradation. This results in a decreased iron efflux from cells and inhibited iron absorption in the duodenum. Hepcidin gene (*HAMP*) expression is stimulated by increased serum and liver iron stores, inflammation, and infection, and it is inhibited by erythropoietic drive and hypoxia. Regulation of *HAMP* expression in response to increased circulating iron levels is maintained by interaction between hemochromatosis (HFE) protein, transferrin receptors (Trf2 and Trf1) and hemojuvelin (Hjv). Increased tissue iron stores are sensed by bone morphogenetic proteins (Bmp6 and Bmp2) binding to ALK2 and ALK3, respectively, and interacting with Hjv. These complexes stimulate SMAD phosphorylation pathway leading to increased *HAMP* transcription [5]. Hereditary hemochromatosis is a group of genetic disorders causing pathological reduction of hepcidin expression leading to body iron overload due to increased iron absorption. The most common mutations, responsible for the majority of hemochromatosis cases in patients with East European ancestry, are mutations in the coding region of *HFE* gene: C282Y rs1800562, H63D rs1799945, and S65C rs1800730. The penetrance of these mutations is modulated by other genetic polymorphisms and unknown factors [6]. The rs855791 C > T mutation in *TMPRSS6* gene encoding transmembrane serine protease matriptase-2, which cleaves multiple proteins from HFE and Bmp6 signaling pathways, reduces enzyme proteolytic activity. This leads to higher *HAMP* expression and lower serum iron, transferrin saturation (TS), and a decreased hemoglobin level in the general population [7,8,9].

Chronic HCV infection is tightly linked with a dysregulation of iron metabolism. Elevated serum iron indices and increased frequency of iron deposits in the liver occur in 30% and in up to 20% of individuals with CHC, respectively. Iron overload in CHC was found to be a significant risk factor for non-response to IFN treatment, hepatic steatosis, liver fibrosis progression, and HCC [10,11]. Phlebotomy treatment of HCV-infected patients significantly reduces liver cancer incidence [12,13]. Molecular pathways leading to dysregulation of iron metabolism in CHC involve direct impact of HCV proteins on the expression of *HAMP* [14]. Therapeutically induced SVR results in normalization of hepcidin levels and serum iron indices [15,16,17]. In CHC patients, markers of serum and hepatocyte iron overload associate with genetic polymorphisms in IFNλ3 gene region, well known predictors of both spontaneous and IFN-treatment-induced HCV clearance [18] as well as *HFE* gene mutations [19]. However, these polymorphisms are not the major factors determining iron overload in HCV-infected subjects and the exact molecular background underlying individual predisposition to the imbalance in iron metabolism in CHC remains unknown [11].

We selected nine relatively common genetic SNP variants related to iron homeostasis (Table S3) and evaluated them in a group of retrospectively analyzed CHC patients. To obtain a deeper insight into the possible mechanisms triggering iron overload in CHC patients, we analyzed the hepatic expression of selected genes involved in iron metabolism. We also measured expression of co-inhibitory receptors which are known to be involved in suppression of immune response in CHC and HCC. We show here that some of the tested polymorphisms are linked with histopathological changes in liver tissue of CHC patients and that *HDAC3* rs976552 together with *CYBRD1* rs884409 may be associated with HCC occurrence.

## 2. Materials and Methods

### 2.1. Patients

Two hundred and forty-nine Polish patients (Caucasian origin) qualified for antiviral treatment with pegylated IFN alpha and ribavirin or DAAs between 2004 and 2014 and with at least a 1.5-year medical record were included in this retrospective study. All patients were treated in the Department of Infectious Diseases, Medical University of Gdansk, Poland. Selected patients were analyzed in previous studies [18,20]. The baseline characteristics of patients are shown in Table S1. Exclusion criteria included: history of drug or alcohol abuse (>25 g alcohol intake/daily), diagnosis of chronic liver diseases other than HCV-related, co-infections HCV/HBV, HCV/HIV. Diagnosis of CHC relied on detection of HCV viremia for at least si months. HCV detection and genotyping was performed as described [21]. The results of histopathological analysis of liver oligobiopsy were available for 211/249 CHC patients. The preparation of liver specimen and classification of inflammation activity, fibrosis and liver iron deposits was previously described [18,22]. The patients were followed for a median of 6.3 years (75% CI 2.9–3.2), calculated by a reverse Kaplan–Meier method [23] (Figure S1). Data on patients’ treatment outcome is summarized in Figure S2. HCC monitoring of all patients was carried according to Polish guidelines [24]. Ultrasound examination of the liver and serum alpha-fetoprotein measurement was carried every 6 months in patients with advanced fibrosis, and every 12 months in those with mild fibrosis. When a tumor was suspected computer tomography or magnetic resonance imaging with contrast was performed.

### 2.2. SNP Genotyping

Genomic DNA was isolated from whole blood samples stored in −80 °C using QIAamp DNA Blood Mini Kit (Qiagen, Hilden, Germany) according to manufacturer instructions. Genotyping of 9 SNPs: *HFE*-rs1800562 G > A C282Y, rs1800730 A > T S65C and rs1799945 C > G H63D, *TFR2* rs7385804 A > C; histone deacetylase 2 (*HDAC2*) rs3778216 C > T; *HDAC3* rs976552 T > G; *HDAC5* rs368328 A > G; *TMPRSS6* rs855791 C > T; duodenal cytochrome b (*CYBRD1*) rs884409 T > G was performed using MassARRAY MALDI-TOF MS platform with MassArray^®^ mass spectrometer (Agena, San Diego, CA, USA) using IPLEX^®^Gold Complete genotyping set with SpectroCHIP^®^ II (Agena, USA) as described [25]. Briefly, the initial PCR amplified nine different products of ~100 bp containing SNPs of interest and a single-nucleotide extension reaction was performed which resulted in allele-specific products of distinct masses. Mass spectra were acquired with a MassARRAY Analyzer 4 mass spectrometer and analyzed with MassARRAY Typer 4.0 software. Amplification and extension primers were designed with Agena Assay Design Suite v2.

### 2.3. Gene Expression Analysis

Total RNA from biopsy liver tissue stored in RNAlater™ Stabilization Solution (Thermo Fisher, Waltham, MA, USA) from 124 CHC patients was isolated using RNeasy Mini Kit (Qiagen, Germany). The cDNA was synthesized with QuantiTect Reverse Transcription Kit (Qiagen, Germany) from 250 ng of total RNA. qRT-PCR amplification was performed using Light Cycler 480 system (Roche Applied Science, Penzberg, Germany) with GUS as a reference gene. We have analyzed expression of genes associated with iron metabolism (*Trf2*, *HAMP*, *Hjv*, *Bmp6*, *Id1*, *HO1*) in 124 liver biopsies. Due to shortage of material mRNA levels of co-inhibitory receptors (*PD-1*, *Tim3*, *CTLA4*) were analyzed in 79 remining biopsy samples. Primer sequences are given in Supporting information (Table S2).

### 2.4. Statistical Analysis

Statistical analysis was carried out using data analysis software STATISTICA version 13 (StatSoft, Inc., Tulsa, OK, USA). All statistical data were presented as a mean ±standard error (SE) or median value (histopathological data). SE was used since the distributions of data were skewed. The analysis was performed using nonparametric statistics: the Mann-Whitney U test, the Chi-square test, Yates’ Chi-square test and Spearman’s rank-order correlation coefficient test. The Bonferroni correction was applied in multiple testing procedures. LD of analyzed SNPs was evaluated using MIDAS software [26]. All statistical tests were 2-tailed. *P* values less than 0.05 were considered statistically significant.

## 3. Results

### 3.1. Histopathological Changes in the Liver 

All SNPs were in Hardy-Weinberg and linkage equilibrium (r^2^ ≤ 0.01). Genotype frequencies are shown in Table S3. The frequency of minor alleles in *HFE* H63D and *TFR2* rs7385804 was significantly higher (*p* < 0.001 for either SNP) in the analyzed group than in the CEU population (TOPMed).

Only two of the tested SNPs associated with dysregulated serum iron indices. Minor allele in *CYBRD1* rs884409 linked with higher serum levels of liver enzymes, increased bilirubin (*p* = 0.009) and iron levels (*p* = 0.011) (Table S3). The presence of mutant allele in *HFE* C2822Y associated with elevated transferrin saturation (*p* = 0.01), ferritin (*p* = 0.004) and higher ALT levels (*p* = 0.02), but it was also more prevalent in men (*p* = 0.013) (Table S3).

Five polymorphisms associated with the presence of histopathological changes in the liver at baseline (Table 1).

### 3.2. HCC Occurrence

HCC diagnosis associated with older age, elevated liver enzymes (*p* ≤ 0.002 for ALT, AST, and GGT), higher iron indices (*p* ≤ 0.01 for sFe, sFerritin, and transferrin saturation), elevated liver inflammation scores at baseline (Table 2), and unsuccessful IFN treatment (*p* = 0.00001). Minor allele in *HDAC3* rs976552 or *CYBRD1* rs884409 linked with HCC occurrence, and the combined effect of minor allele in both SNPs was even stronger (Table 2). No other polymorphism associated with HCC.

**Figure 1:**
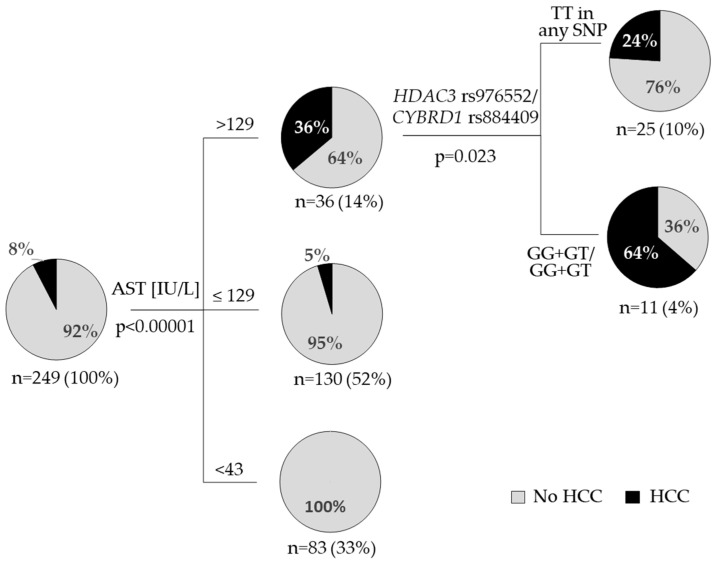
Decision tree model of HCC occurrence, calculated by the CHAID algorithm. The hierarchy model included all significant factors associated with HCC (Table 2): age, all liver enzymes, serum iron parameters, *HDAC3* rs976552 and *CYBRD1* rs884409 (individual and combined effect of minor alleles). Pie charts indicate the rate (%) of HCC in each group of patients. The number of patients and the percentage of the overall population are indicated. *P* values are corrected.

Multivariate logistic regression model of HCC occurrence was constructed by backward stepwise regression with all variables significant in monovariate logistic regression analysis at the input (Table S4). Only AST, sFe, and ALT were automatically selected by the algorithm (validated AUCROC of 0.738; Table S4) and this model was only slightly improved when the presence of the minor allele in *HDAC3* rs976552/*CYBRD1* rs884409 was added for comparison (validated AUCROC 0.839; Figure S3; Table S4). On the other hand, minor allele status in *HDAC3* rs976552/*CYBRD1* rs884409 together with AST were the most informative variables in the decision tree model of HCC occurrence built with the CHAID (Chi-squared Automatic Interaction Detector) method (Figure 1). 

### 3.3. Hepatic Gene Expression 

Polymorphism in *HDAC3* associated with hepatic expression of *CTLA4* gene but not *PD-1* or *Tim3* mRNA levels (Figure 1). Patients with both dominant T alleles had significantly higher *CTLA4* expression levels (TT vs. GT + GG, *p* = 0.01) No significant correlation between *CYBRD1 rs884409* polymorphism, minor allele status in both *HDAC3 rs976552/CYBRD1 rs884409* and gene expression were found (Figure S4). 

Patients with *HFE* S65C mutation had higher liver hepcidin expression (*p* = 0.029) (Table S6). No other associations between gene expression and analyzed polymorphisms were identified. In patients with C282Y rs1800562 mutation ratios of expression of *Trf2*, *Hjv*, *Bmp6* and *HO1*, but not *HAMP* and *Id1*, normalized to either sFerritin or sFe were lower than in the rest of individuals. *HFE* S65C mutation decreased *HAMP*/sFe and *Id1*/sFe ratios, while *HFE* H63D polymorphism was not associated with any changes in these parameters (Table S6). 

## 4. Discussion

Among SNPs selected according to their impact on iron metabolism only *HFE* C282Y and *CYBRD1* rs884409 associated with serum iron indices in CHC patients. There was no correlation between tested SNPs and hepatic expression of genes involved in iron regulation, except for *HFE* S65C polymorphism which associated with *HAMP* expression (Table S5). In order to better reflect regulation of iron metabolism in condition of liver inflammation in CHC we have normalized expression levels of iron-related genes to serum iron or ferritin concentrations to correct for iron stores. Our analysis revealed differential impact of *HFE* polymorphisms on iron metabolic genes in CHC (Table S5) which may directly result from structural differences between mutated *HFE* proteins. The C282Y rs1800562 mutation disrupts a critical disulfide bond in the α3 domain of HFE limiting its localization mostly to the cytoplasm. Two others SNPs, namely H63D rs1799945 and S65C rs1800730, affect the α1 binding groove of HFE protein, which interacts with Tfr1, but do not change cellular HFE localization. Surprisingly we found higher *HAMP* expression in patients with S65C allele but the ratio of *HAMP*/sFe was lower for these individuals (Table S5), suggesting that regulation of *HAMP* expression in CHC is mainly driven by inflammation, as it was shown previously [18,27]. Our results are consistent with other reports showing that regulation of iron metabolism in CHC is largely dependent on HCV-mediated inflammation and patients’ immune status [15,18]. We also show here that SNPs involved in normal iron regulation may contribute to liver disease in CHC (Table 1). However, their impact on the progression of hepatic pathology still needs to be verified. This is probably due to the fact that iron status reciprocally modulates immune function in inflammatory diseases [28]. 

In our study, carriers of two dominant alleles in H63D rs1799945 were less frequently diagnosed with hepatic iron overload (Table 1). The role of *HFE* mutations in secondary iron overload diagnosed in CHC still remains unclear, as some studies show association H63D and C282Y mutations with elevated serum iron indices [19,29], liver iron deposits [11], and others reported no such relation [6,30]. Our results confirm the notion that HFE mutations may contribute to (but do not fully explain) hepatic iron accumulation in chronic hepatitis C.

The occurrence of HCC during follow up was associated with elevated serum iron indices (Table 2). Our result confirms the well-known association of iron overload in CHC with liver inflammation and HCC [10,11,12,13]. Additionally, we found higher prevalence of HCC in the group of carriers of two minor alleles: *HDAC3* rs976552 G and *CYBRD1* rs884409 G (Table 2, Figure 1). Decision tree model showed that this discriminating effect was the most significant in the group of patients with elevated AST (>129 IU/L) (Figure 1). To our knowledge we show this relation here for the first time, but it agrees with existing literature data connecting HDACs and *CYBRD1* with liver pathology and carcinogenesis. Histone deacetylases (HDACs) regulate variety of cellular processes including key innate immune pathways and stimulation of antiviral responses through IFNα signaling [31]. HCV infection increases cellular activity of HDAC3 which leads to suppressed *HAMP* expression [32,33]. HDAC3 was shown to impact hepatic steatosis [34]. Polymorphisms in *HDAC2* rs3778216, HDAC3 rs976552, and *HDAC5* rs368328 were independent predictors of treatment outcome in CHC patients treated with peg-IFNa with ribavirin and improved the predictive value of SNP in *IL-28B* gene [35]. Expression of HDACs is elevated in HCC and can predict tumor recurrence and survival of these patients [36,37]. Application of selective HDAC inhibitors was found to be a promising strategy in HCC treatment [38].

CYBRD1 is a ferroreductase involved in the absorption of iron in the duodenum. The *CYBRD1* rs884409 polymorphism associates with variation in serum transferrin saturation and ferritin concentration in patients with *HFE* C282Y mutation and the mutated allele reduces *CYBRD1* promoter activity by 30% [39,40]. *CYBRD1* rs884409 was found previously to associate with liver inflammation, liver enzymes and bilirubin in CHC patients [20]. In our study this SNP was linked with liver inflammation grade (Table S4) and liver steatosis (Table 1, Table S5) as well as serum liver enzymes, bilirubin, and iron concentration (Table S6). Apart from iron metabolism CYBRD1 is involved in other cellular functions which are not fully understood. The link between major allele in *CYBRD1* rs884409 and lower frequency of HCC occurrence found in our study agrees with reports showing correlation of elevated *CYBRD1* expression with a diminished proliferation, invasion and adhesion of cancer cells [41,42] as well as prolonged recurrence-free survival in treated breast cancer patients [42]. Additionally, CYBRD1 inhibited activation of focal adhesion kinase which is crucial for tumor adhesion and metastasis and this effect was independent of cellular iron signaling pathways [41,42].

Cytotoxic T lymphocyte-associated antigen (CTLA4), programmed cell death 1 (PD-1), Tim3 (T cell immunoglobulin and mucin-domain containing protein 3) are immune checkpoint receptors that deliver inhibitory signals to T cells and are highly expressed during CHC on exhausted and dysfunctional T cells [43,44,45]. Additionally, they are commonly overexpressed in many cancer types including HCC, and immune checkpoint inhibitors are intensively studied as cancer immunotherapy [46]. PD-1 and Tim-3 are threshold receptors which are already expressed on the surface of cells while CTLA4 is a negative feedback receptor upregulated on T cells upon activation but constitutively expressed on Tregs [47,48]. The fundamental role of CTLA4 is the inhibition of T cell activation, by competing with CD28 binding to its ligands, which occurs during initiation of the immune response [49].

**Figure 2:**
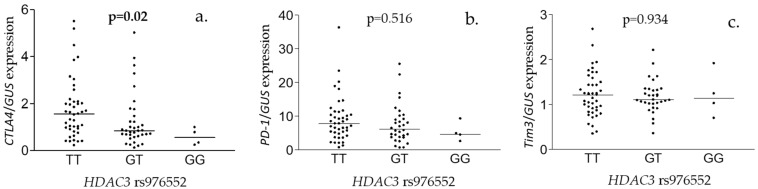
*HDAC3* rs976552 and hepatic expression of co-inhibitory receptors. Hepatic expression of *CTLA4* (**a**), *PD-1* (**b**), and *Tim3* (**c**) in samples from CHC patients with different *HDAC3* rs976552 genotypes. Shown are the *p* values from a Kruskal–Wallis test.

Interestingly we found that major allele in *HDAC3* rs976552, which also linked with lower number of HCC cases, associated with higher hepatic expression of *CTLA4* (Figure 2). Additionally, *CTLA4* expression positively correlated with ALT, AST, bilirubin, sFe, transferrin saturation, sFerritin and liver inflammation (Table S7), and patients homozygotic for minor *HDAC3* allele had lower AST levels (Table S5). This apparent inconsistency could be explained by the fact that higher *CTLA4* expression in the early stages of HCV infection may be favorable for disease course as it could protect from overactivation of the immune response in the liver, but at the same time indicate robust immune response to infection [49]. We have previously reported that CHC patients with IL-28 rs12979860 CC genotype which is favorable for disease outcome exhibited higher liver inflammation at the time of qualification [18]. Additionally, CTLA4 action in T cells is regulated on the translational level and by trafficking from intracellular vesicles to the cell membrane and endocytosis [50]. It is possible that liver inflammation and elevated liver enzymes are reflection of different opposing effects of immune reaction to HCV infection. To dissect these processes, it would be necessary to monitor liver disease progression and hepatic *CTLA4* expression over time. We hypothesize that elevated *CTLA4* expression could indicate early stage of inflammation which is accompanied by a flare of liver enzymes and an ongoing process of inhibition of overt T-cell activation. The link we observed between polymorphisms in *HDAC3* and *CTLA4* expression is supported by the study in which HDAC inhibitor Belinostat was found to potentiate the antitumor effect of anti-CTLA4 antibody in subcutaneous murine model of HCC [51].

The strong point of our study is a relatively large number of analyzed liver biopsy samples as well as a long follow up time. Our study also has some limitations. We could not verify if hepatic expression of inhibitory receptors at baseline is linked with cancer occurrence during follow up due to a small group of patients with HCC and liver biopsy samples available for analysis. Lack of paired liver biopsies from the end of observation made it impossible to evaluate the rate at which liver disease progression occurred in these patients. Also, we were unable to determine the true duration of HCV infection as diagnostic and screening protocols were not fully developed in 2004 in comparison to the year 2014. Our results on the association of genetic background with HCC occurrence certainly need to be evaluated on a larger cohort of patients. Especially it would be interesting to verify the link between hepatic expression of co-inhibitory receptors, genetic variation in *HDAC3* and *CYBRD1,* and liver disease progression to HCC. Due to the current diagnostic protocols where the liver biopsy is rarely needed, such studies would probably have to be performed on an animal model.

## 5. Conclusions

Polymorphisms in genes associated with iron homeostasis associate with liver disease in CHC but are not the crucial factors determining secondary iron overload. The presence of minor allele in both *HDAC3* rs976552/*CYBRD1* rs884409 is linked with higher prevalence of HCC in these patients, especially in the group of patients with significantly elevated AST (>129 IU/L). Unfavorable G allele in *HDAC3* rs976552 associates with lower hepatic expression of immune checkpoint receptor *CTLA4* mRNA. Further studies are needed to determine the significance of HDAC3 and CYBRD1 in liver disease progression to HCC in CHC patients.

## Figures and Tables

**Table 1:** Association between selected SNPs and the presence of histopathological changes in the liver.

Genotype		Steatosis		Advanced Fibrosis *		Iron Deposits	
		OR (CI 95%)	*p*	OR (CI 95%)	*p*	OR (CI 95%)	*p*
*CYBRD1* rs884409	TT (*n* = 140)	0.5 (0.3–0.9)	0.029	0.7 (0.4–1.4)	NS	1.0 (0.6–1.9)	NS
	GG (*n* = 12)	2.0 (0.5–7.6)	NS	3.7 (17.8–0.8)	NS	1.0 (0.3–3.6)	NS
*HDAC5* rs368328	AA (*n* = 96)	2.3 (1.3–4.1)	0.006	2.2 (1.2–4.1)	0.010	1.1 (0.6–2.0)	NS
	GG (*n* = 26)	0.5 (0.2–1.1)	NS	0.6 (0.3–1.3)	NS	0.8 (0.3–1.9)	NS
*TFR2* rs7385804	AA (*n* = 58)	1.0 (0.5–1.8)	NS	0.6 (0.3–1.2)	NS	0.9 (0.4–1.8)	NS
	CC (*n* = 42)	0.8 (0.4–1.6)	NS	2.4 (1.1–5.3)	0.035	0.6 (0.3–1.2)	NS
*TMPRSS6* rs855791	CC (*n* = 83)	0.8 (0.5–1.4)	NS	0.5 (0.3–0.8)	0.011	1.5 (0.8–2.7)	NS
	TT (*n* = 20)	0.9 (0.4–2.4)	NS	3.6 (1.1–11.8)	NS	0.5 (0.2–1.4)	NS
*HFE* H63D rs1799945	CC (*n* = 144)	0.8 (0.4–1.4)	NS	0.7 (0.4–1.2)	NS	0.5 (0.2–0.9)	0.022
	GG (*n* = 6)	0.6 (0.1–3.0)	NS	0.5 (0.1–2.0)	NS	0.7 (0.1–3.7)	NS

Results of multivariate logistic regression analyses, adjusted for age and sex; OR-odds ratio; CI—confidence intervals; NS—not significant; * advanced fibrosis ≥ 2 on a 0–4 grading scale.

**Table 2:** Baseline characteristics of patients diagnosed with HCC during the follow-up period.

Characteristisc (*n* = 249)		HCC		*p*	OR (CI 95%)
		No (*n* = 230)	Yes (*n* = 19)		
Age [yr]		47 ± 1	53 ± 2	0.022	
Sex (Male/Female)		140/90	13/6	0.686	
HGB [g/dL]		14.8 ± 0.1	14.6 ± 0.4	0.579	
ALT [IU/L]		109 ± 6	187 ± 23	0.0002	
AST [IU/L]		72 ± 3	164 ± 19	<0.00001	
GGT [IU/L]		97 ± 6	152 ± 20	0.002	
Bilirubin [mg/dL]		0.8 ± 0.04	1.2 ± 0.1	0.0006	
sFe [μg/dL]		147 ± 4	215 ± 15	0.00004	
Transferrin saturation [%]		42 ± 1	67 ± 10	0.010	
sFerritin [ng/mL]		351 ± 31	609 ± 89	0.002	
Histopathology *n* = 211		No (*n* = 97)	Yes (*n* = 14)	*p*	
Inflammation grade		2 (2/2)	3 (2/3)	0.001	
Fibrosis grade		2 (1/3)	3 (2/3)	0.276	
Iron deposits grade (0–3)		0 (0/1)	0 (0/1)	0.618	
Steatosis grade (0–3)		1 (0/2)	1 (0/2)	0.719	
Hepatocyte iron deposits present (yes/no)		70/127	6/8	0.621	
Hepatocyte steatosis present (yes/no)		118/79	8/6	0.937	
Liver fibrosis present (yes/no)		119/78	12/2	0.050	
Polymorphism *n* = 249		No (*n* = 230)	Yes (*n* = 19)	*p*	
*HDAC3* rs976552	TT	155 (96%)	7 (4%)	0.026 ^#^	
	GT	70 (86%)	11 (14%)		
	GG	5 (83%)	1 (17%)		
	TT	155 (96%)	7 (4%)	0.011 *	3.6 (1.3–9.8)
	GG + GT	75 (86%)	12 (14%)		
*CYBRD1* rs884409	TT	155 (95%)	8 (5%)	0.041 *	2.5 (1.0–7.1)
	GT + GG	75 (87%)	11 (13%)		
*HDAC3* rs976552/ *CYBRD1* rs884409	TT in any SNP	203 (95%)	10 (5%)	0.001 *	8.1 (2.2–29.2)
	GG + GT/GG + GT	27 (75%)	9 (25%)		

Quantitative biochemical data is shown as mean ± SE; data for inflammation, fibrosis, iron deposits and steatosis is shown as median values with percen^ti^les ^(^25th/75th). ^#^ *p* for log-linear analysis of main effect, * *p* for multivariate logistic regression analysis (adjusted for age and sex). sFe, serum iron; sFerritin, serum ferritin.

## Data Availability

The data presented in this study is contained within the article and Supplementary Material.

## Supplementary Materials

The following supporting information can be downloaded at: https://www.mdpi.com/article/10.3390/v15081710/s1.
