# Supplementary material for: Polymorphisms Related to Iron Homeostasis Associate with Liver Disease in Chronic Hepatitis C
Source: Viruses. 2023 Aug 9;15(8):1710. doi: 10.3390/v15081710 (PMC10457817; doi:10.3390/v15081710)
Supplement: Supplementary file 1 [file viruses-15-01710-s001.zip › viruses-2501162-supplementary.pdf]

Supplementary data

Polymorphisms Related to Iron Homeostasis Associate with Liver Disease in Chronic Hepatitis C

Anna Wróblewska, Anna Woziwodzka, Magda Rybicka-Misiejko, Krzysztof P. Bielawski, Katarzyna Sikorska

Table S1. Characteristics of patients selected for the study………………………………….……… 2

Figure S1. Reverse Kaplan-Meier curve showing patients follow up time………………………… 3

Figure S2. Summary of the therapeutic outcome for patients included in the study…………….. 4

Table S2. Primers used for quantitative gene expression analysis…………………………………... 5

Table S3. Genotype distribution within single-nucleotide polymorphisms selected for the study in chronic hepatitis C patients……………………………………………………………………………… 6

Table S4. Construction of logistic regression model for HCC occurrence…………………………. 7

Figure S3. ROC curves for validated logistic regression models for HCC occurrence……………. 8

Table S5. Polymorphisms associated with biochemical and histopathological data……………… 9

Table S6. SNPs associated with hepatic iron-related gene expression normalized to serum iron indices…………………………………………………………………………………………………… 10

Table S7. Spearman rank correlation coefficients for associations between hepatic gene expression and baseline biochemical parameters for CHC patients …..…………………….…..…..11

Figure S4. Hepatic expression of co-inhibitory receptors in relation to selected genotypes……….12

Table S1. Characteristics of patients selected for the study.

Variable	Characteristics
Gender (Male/Female)	153/96
Age (yr)	19 - 78 (50)
Hemoglobin (g/dL)	9.6 – 18.4 (14.9)
ALT (IU/L)	14-852 (79)
AST (IU/L)	3-369 (59.5)
GGT (IU/L)	9-663 (70)
Bilirubin (mg/dL)	0.2-4.2 (0.7)
Iron (μg/dL)	29-357 (146)
Transferrin saturation (%)	6-100 (40)
Ferritin (ng/mL)	7-3410 (218)
Inflammation grade (0-3)	2 (2/2)
Fibrosis grade (0-4)	2 (1/3)
Iron deposits grade (0-3)	0 (0/1)
Steatosis grade (0-3)	1 (0/2)
HCV genotype (n=131)	120 genotype 1
	15 genotype 3
	9 genotype 4
HCV RNA (kIU/mL) (n=89)	37-24047 (1390)
Median observation time [years]	6.1 (min-max 1.5-20)
Median follow up time [years]	6.3 (75% CI 2.9-9.3)
HCC yes/no	19/230

Quantitative variables are presented as minimal - maximal values (median). Histopathological data is shown as median values with percentiles (25th/ 75th).

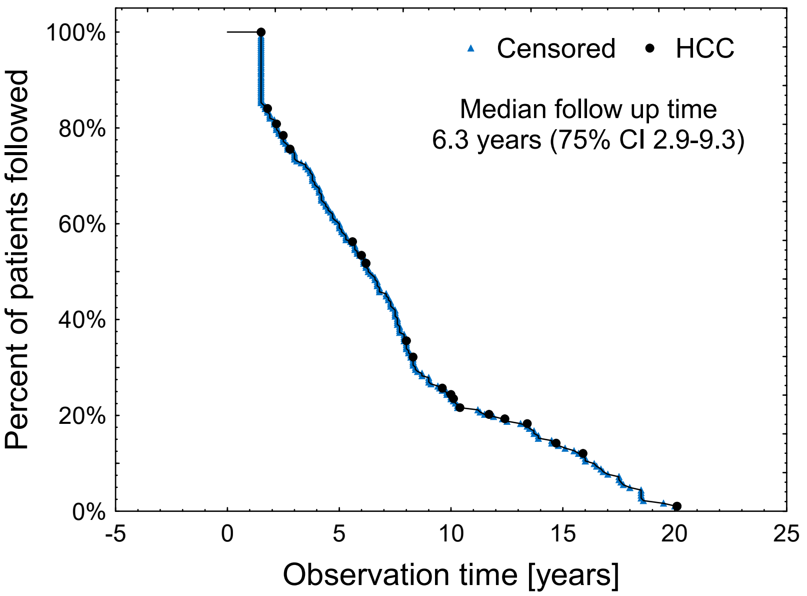

Figure S1. Reverse Kaplan-Meier curve showing patients follow up time.

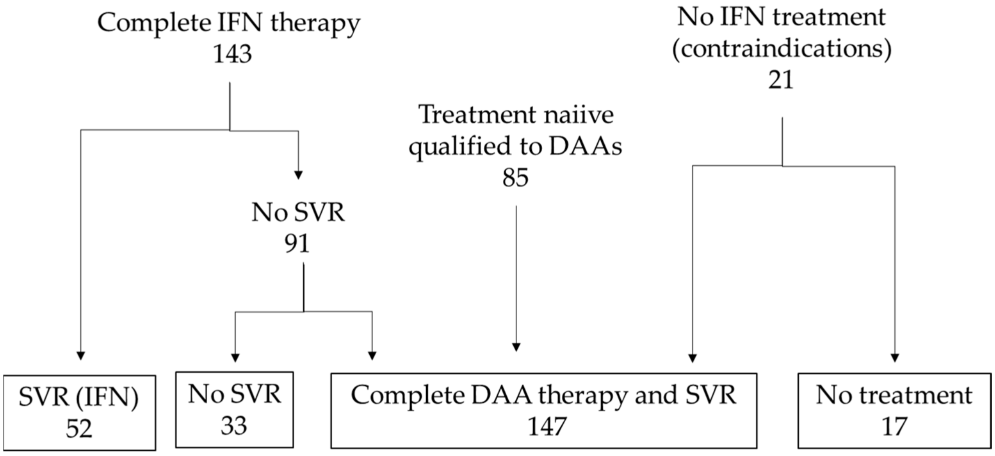

Figure S2. Summary of the therapeutic outcome for patients included in the study.

Table S2. Primers used for quantitative gene expression analysis.

**Primer name**	**Primer sequence 5′-->3′**	**Reference**
GUS_F	CGAGAGTGCTGGGGAATAAA	[18]
GUS_R	CCTGGTTTCATTGGCAATCT	
HAMP_F	AGACACCCACTTCCCCATCT	[18]
HAMP_R	CACATCCCACACTTTGATCG	
FPN1_F	CAGGGACTGAGTGGTTCCAT	[18]
FPN1_R	ACCACATTTTCGACGTAGCC	
HJV_F	GGCCTAGGAGACACGTGAAA	[20]
HJV_R	GAGGCTGGAAAAATTGGTGA	
TFR2_F	GACCCTGCAGTGGGTGTACT	[20]
TFR2_F	CAGTCGCTCGTCTCTCTCCT	
HO-1_F	GAAAAGCACATCCAGGCAAT	[20]
HO-1_R	CTGCTGCAGGAACTGAGGAT	
ID1_F	TGTTCCATTTTCCGTATCTGC	[20]
ID1_R	TGAAACAGAATGGGCAAAGC	
BMP6_F	CTTACGACAAGCAGCCCTTC	[20]
BMP6_R	CACGTGCACCTCACTCACTT	
CTLA4_F	CTGTGCGGCAACCTACATGA	this study
CTLA4_R	TGCAGATGTAGAGTCCCGTG	
PD-1_F	GCCTGTGTTCTCTGTGGACT	this study
PD-1_R	ATGGTGGCATACTCCGTCTG	
Tim3_F	GGAGCCTGTCCTGTGTTTGA	this study
Tim3_R	AGGGACACATCTCCTTTGCG	

Table S3. Genotype distribution within single-nucleotide polymorphisms selected for the study in chronic hepatitis C patients.

No	Gene	dbSNP ID / mutation	SNP associated with	Consequence type	MAF (TOPMed)	Clinical significance	Ref.	Genotype	No of cases #	Freq.#
1	Hemochromatosis (*HFE*)	rs1800562 G>A / C282Y	serum transferrin and sFe in healthy individuals	missense variant (different aa sequence), 2KB upstream variant	A=0.053	Hereditary hemochromatosis; complications of diabetes, Alzheimer disease; familial porphyria cutanea tarda	[6]	GG	233	0.94
								GA	16	0.06
								AA	0	0
2		rs1800730 A>T / S65C		missense variant, non coding transcript variant	T=0.009			AA	241	0.97
								AT	8	0.03
								TT	0	0
3		rs1799945 C>G / H63D			G=0.100			CC	172	0.7
								CG	68	0.3
								GG	9	0.04
4	Transferrin receptor 2 (*TFR2*)	rs7385804 A>C	sFe levels in healthy individuals	Intron variant	C=0.334	not reported in ClinVar	[7]	AA	71	0.3
								AC	128	0.5
								CC	50	0.2
5	Histone deacetylase 2 (*HDAC2*)	rs3778216 C>T	hepcidin expression; cellular iron metabolism, inflammatory response	Intron variant	T=0.218	not reported in ClinVar	[34,36]	CC	132	0.5
								CT	104	0.4
								TT	13	0.1
6	Histone deacetylase 3 (*HDAC3*)	rs976552 T>G		2KB Upstream variant	C=0.210	not reported in ClinVar		TT	162	0.7
								TG	81	0.3
								GG	6	0.02
7	Histone deacetylase 5 (*HDAC5*)	rs368328 A>G		Intron variant	G=0.328	not reported in ClinVar		AA	109	0.4
								AG	112	0.4
								GG	28	0.1
8	Transmembrane serine proteinase 2, matriptase-2 (*TMPRSS6*)	rs855791 C> T	sFe, TS; blood hepcidin mRNA and protein in urine in healthy individuals	missense variant	A=0.361	microcytic anemia, iron-refractory iron deficiency anemia	[8-9]	CC	92	0.4
								CT	128	0.5
								TT	29	0.1
9	Duodenal cytochrome b (*CYBRD1*)	rs884409 T>G	serum TS and ferritin concentration	2 KB upstream sequence variant	G=0.218	not reported in ClinVar	[20,40-41]	TT	163	0.6
								TG	73	0.3
								GG	13	0.04

sFe -serum iron; TS – transferrin saturation; MAF (TOPMed) – minor allele frequency from TOPMed Programme; #this study

Table S4. Construction of logistic regression model for HCC occurrence.

**Input variables**	**p***	**Multivariate logistic regression model**					
		**Model no**	**Variables in the model**	**Training set**		**Cross-validated (10-fold)**	
				AUC	AUC error	AUC	AUC error
AST	<0.000001	**1**	AST	0.842	0.05	0.808	0.06
sFe	0.00009	**2**	AST+sFe	0.850	0.05	0.827	0.05
Minor *HDAC3* rs976552/ *CYBRD1* rs884409	0.0003	**3**	AST+sFe+ALT	0.883	0.07	0.738	0.11
Liver inflammation grade	0.0003	**4**	AST+sFe+ALT+ Minor *HDAC3* rs976552/ *CYBRD1* rs884409	0.871	0.05	0.839	0.06
TS	0.0009						
ALT	0.004						
Age	0.021						
Billirubin	0.023						
GGT	0.039						
sFerritin	0.047						

* p values for monovariate logistic regression analysis. **Model 3** was automatically constructed using backward stepwise regression with all the listed variables significant in the monovariate analysis as an input. **Model 1**, **Model 2** and **Model 4** were evaluated for comparison.

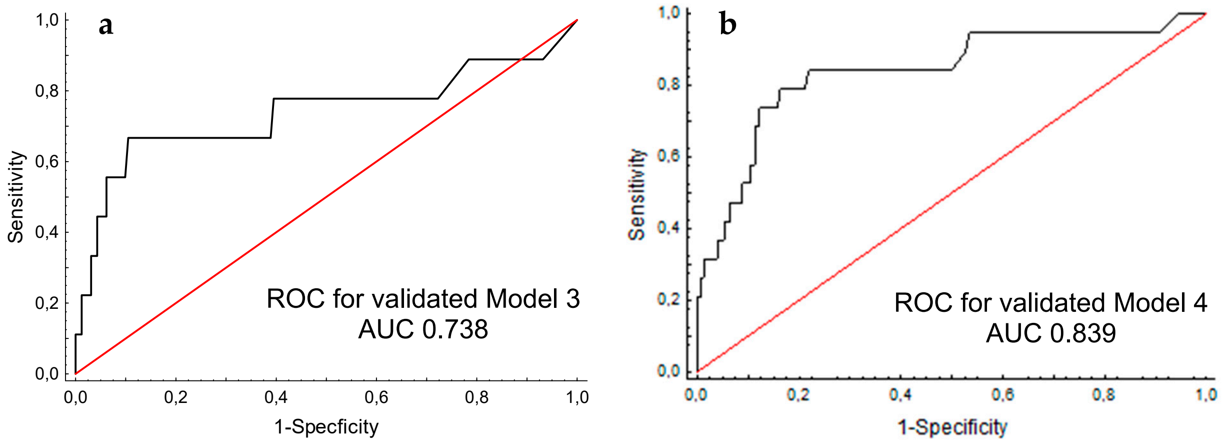

Figure S3. ROC curves for validated logistic regression models for HCC occurrence. Model 3 (**a**) was generated automatically using backward stepwise regression with all the variables significant in the monovariate analysis as an input, and Model 4 (**b**) contained additionally minor allele status in *HDAC3* rs976552/ *CYBRD1* rs884409 for comparison.

Table S5. Polymorphisms associated with biochemical and histopathological data.

**Parameter**	**Polymorphism**														
	***HFE* C282Y rs1800562 G>A**			***HFE* H63D rs1799945 C>G**			***HDAC3* rs976552 T>G**			***CYBRD1* rs884409 T>G**			**Minor *HDAC3* rs976552/ *CYBRD1* rs884409**		
	**GG n=233**	**GA n=16**	**p**	**CC+CG n=240**	**GG n=9**	**p**	**TT+GT n=243**	**GG n=6**	**p**	**TT n=163**	**GG+GT n=86**	**p**	**TT in any SNP n=213**	**GG+GT/ GG+GT n=36**	**p**
Age [yr]	47±1	52±2	0.172	48±1	52±2	0.337	48±1	41±7	0.391	47±1	49±1	0.694	48±1	46±2	0.175
Sex (Male/Female)	**138/95**	**15/1**	**0.013**	90/150	6/3	0.436	150/93	3/3	0.874	99/64	54/32	0.751	83/130	13/23	0.872
HGB [g/dL]	14.7±0.1	15.4±0.3	0.082	14.8±0.1	14.0±0.6	0.291	14.7±0.1	15.1±0.8	0.577	14.8±0.1	14.6±0.2	0.237	14.7±0.1	14.8±0.3	0.858
ALT [IU/L]	**111±6.2**	**167±27**	**0.019**	112±6	170±35	0.050	116±6	57±12	0.064	**106±7**	**131±11**	**0.036**	111±7	139±16	0.079
AST [IU/L]	79±4	91±16	0.382	**78±4**	**123±22**	**0.016**	**80±4**	**39±7**	**0.035**	**69±4**	**98±8**	**0.002**	**74±4**	**111±13**	**0.002**
GGT [IU/L]	101±6	109±19	0.226	103±6	58±12	0.189	102±6	85±116	0.372	**87±6**	**129±13**	**0.016**	97±7	129±17	0.127
Bilirubin [mg/dL]	0.9±0.04	0.8±0.1	0.925	0.9±0.04	0.8±0.1	0.439	0.9±0.04	0.6±0.1	0.142	**0.8±0.04**	**1.0±0.1**	**0.009**	0.8±0.04	0.9±0.1	0.769
Serum iron [µg/dL]	151±5	179±13	0.067	151±4	192±29	0.152	153±4	125±14	0.392	**145±5**	**167±7**	**0.011**	150±5	167±12	0.161
Transferrin saturation [%]	**42±1.5**	**56±6**	**0.011**	43±2	51±9	0.339	43±2	34±3	0.330	40±2	48±3	0.078	42±2	46±5	0.764
Ferritin [ng/mL]	**348±27**	**708±220**	**0.004**	372±30	339±95	0.998	375±30	117±48	0.126	343±35	420±52	0.170	366±33	394±63	0.710
**Histopathology n=211**	n=197	n=14	p	n=205	n=6	p	n=206	n=5	p	n=140	n=71	p	n=181	n=30	p
Inflammation grade (0-3)	2(1/2)	2(2/3)	0.150	2(2/2)	2(1.5/2)	0.818	2(2/2)	2(2/2)	0.718	**2(1/2)**	**2(2/3)**	**0.013**	**2(1/2)**	**2(2/3)**	**0.021**
Fibrosis grade (0-4)	2(1/3)	2(1/3)	0.842	2(1/3)	1.5(1/2)	0.289	2(1/3)	1(1/2)	0.277	2(1/3)	2(1/3)	0.095	2(1/3)	2(2/3)	0.191
Iron deposits grade (0-3)	1(0/1)	1(0/2)	0.154	0(0/1)	0(0/1)	0.761	0(0/1)	0(0/0)	0.179	0(0/1)	0(0/1)	0.890	0(0/1)	0(0/1)	0.698
Steatosis grade (0-3)	1(0/2)	2(0/2)	0.292	1(0/2)	0.5(0/2)	0.772	1(0/2)	2(0/2)	0.832	1(0/2)	1(0/2)	0.292	1(0/2)	1(0/2)	0.610
Hepatocyte iron deposits present (yes/no)	68/129	8/6	0.161	75/130	1/5	0.871	76/130	0/5	0.217	50/90	26/45	0.926	65/116	11/19	0.883
Hepatocyte steatosis present (yes/no)	117/80	9/5	0.937	123/82	3/3	0.944	123/83	3/2	0.654	**76/64**	**50/21**	**0.023**	107/74	19/11	0.814
Liver fibrosis present (yes/no)	122/75	9/5	0.913	128/77	3/3	0.848	129/77	2/3	0.573	82/58	49/22	0.140	110/71	21/9	0.446

Quantitative biochemical data is shown as mean ± SE; histopathological data is shown as median values with percentiles (25th/ 75th).

Table S6. SNPs associated with hepatic iron-related gene expression normalized to serum iron indices.

**Parameter**	*HFE* C282Y rs1800562 G>A			*HFE* S65C rs1800730 A>T			*HFE* H63D rs1799945 C>G			*HDAC2* rs3778216 C>T			*TMPRSSR6* rs855791 C>T		
	GG n=233	GA n=16	p	AA n=241	AT n=8	p	CC n=172	GC+GG n=77	p	CC n=132	CT+TT n=117	p	CC+CT n=220	TT n=29	p
sFerritin	**347±27**	**707±221**	**0.004**	367±30	459±150	ns	388±39	331±35	ns	155±6	149±7	ns	381±32	289±60	ns
sFe	150±5	178±13	ns	152±4	150±38	ns	149±5	161±8	ns	410±45	323±34	ns	154±5	141±12	ns
**Gene expression**	n=116	n=8		n=118	n=6		n=85	n=39		n=71	n=53		n=111	n=13	
HAMP	1.6±0.1	1.9±0.3	ns	**1.6±0.1**	**2.9±0.6**	**0.029**	1.9±0.3	1.5±0.1	ns	1.8±0.1	1.6±0.2	ns	1.7±0.1	1.6±0.3	ns
FPN1	14±1	10±2	ns	14±0.8	13±3.4	**ns**	13±1	14±2	ns	13±1.0	15±1.3	ns	14±1	11±2	ns
**Ratio***															
Tfr2/ sFerritin	**13±2**	**3±1**	**0.008**	5±1	12±2	ns	11±4	13±2	ns	10±2	16±4	ns	12±2	11±3	ns
HAMP/ sFerritin	9±2	4±1	ns	8±2	9±2	ns	6±1	10±2	ns	9±2	8±2	ns	9±2	11±3	ns
Hjv/ sFerritin	**10±2**	**2±1**	**0.007**	4±1	10±2	ns	8±2	10±2	ns	**7±1**	**13±3**	**0.046**	9±3	11±4	ns
Bmp6/ sFerritin	**10±1**	**2±1**	**0.010**	4±1	10±1	ns	7±1	11±2	ns	**7±1**	**12±2**	**0.049**	9±1	11±4	ns
Id1/ sFerritin	10±1	6±3	ns	6±1	10±1	ns	8±1	10±1	ns	9±1	10±1	ns	9±1	13±4	ns
HO1/ sFerritin	**17±2**	**4±1**	**0.017**	6±1	16±2	ns	14±1	17±3	ns	**12±2**	**21±4**	**0.030**	16±2	16±4	ns
FPN1/sFerritin	**2±0.4**	**0.3±0.1**	**0.008**	2±0.3	0.6±0.1	ns	1.8±0.4	1.7±0.7	ns	1.3±0.2	2.4±0.7	ns	1.8±0.4	1.7±0.6	ns
Tfr2/ sFe	**9±1**	**5±1**	**0.009**	14±5	8±1	ns	8±1	9±1	ns	8±1	9±1	ns	9±1	9±1	ns
HAMP/ sFe	9±2	7±1	ns	**23±7**	**8±1**	**0.001**	9±1	9±1	ns	9±1	8±1	ns	9±1	10±2	ns
Hjv/ sFe	**7±0.5**	**4±0.3**	**0.006**	10±3	6±0.4	ns	6±1	7±1	ns	6±1	6±1	ns	**6±0.5**	**8±1**	**0.022**
Bmp6/ sFe	**7±1**	**4±1**	**0.016**	14±6	7±1	ns	6±0.4	8±1	ns	6±0.5	5±1	ns	**7±1**	**8±1**	**0.031**
Id1/ sFe	10±1	9±2	ns	**18±4**	**9±1**	**0.021**	9±1	10±1	ns	10±1	9±1	ns	10±1	10±2	ns
HO1/ sFe	**13±1**	**8±1**	**0.034**	17±4	12±1	ns	11±1	13±1	ns	11±1	15±2	ns	12±1	13±2	ns
FPN1/sFe	**1.4±0.1**	**0.7±0.2**	**0.041**	1.3±0.1	1.4±0.3	ns	1.3±0.1	1.4±0.2	ns	1.2±0.2	1.5±0.2	ns	1.3±0.1	1.3±0.3	ns

*ratio of relative gene expression in liver biopsy normalized to serum serum ferritin (sFerritin) or serum iron (sFe)

Table S7. Spearman rank correlation coefficients for associations between hepatic gene expression and baseline biochemical parameters for CHC patients.

Relative gene expression	Parameter								
	**Age**	**ALT**	**AST**	**GGT**	**Bilirubin**	**sFe**	**TS**	**sFerritin**	**Liver inflammation**
Tfr2	ns	ns	ns	ns	ns	ns	ns	ns	ns
HAMP	0.196[d]	ns	ns	0.221[d]	ns	0.394[a]	0.467[a]	0.641[a]	ns
Hjv	-0.201[d]	ns	-0.223[d]	ns	ns	ns	ns	ns	-0.293[c]
Bmp6	-0.214[d]	ns	ns	ns	ns	ns	ns	ns	ns
Id1	0.282[d]	0.245[d]	0.244[d]	0.319[c]	0.240[d]	0.492[a]	0.548[a]	0.595[a]	ns
HO1	ns	0.308[c]	0.319[c]	0.262[d]	0.239[d]	0.207[d]	ns	0.292[d]	ns
FPN1	ns	ns	ns	ns	ns	ns	ns	ns	ns
Tim3	ns	ns	ns	ns	ns	ns	ns	ns	ns
PD-1	ns	0.462[b]	0.422[c]	ns	0.290[d]	0.247[d]	ns	ns	0.267[d]
CTLA4	ns	0.557[a]	0.526[a]	ns	0.279[d]	0.304[d]	0.246[d]	0.230[d]	0.301[d]

[a], $p<0.00001$; [b], $p<0.0001$; [c], $p<0.001$; [d], $p<0.05$; ns, not significant; sFe, serum iron; sFerritin, serum ferritin.

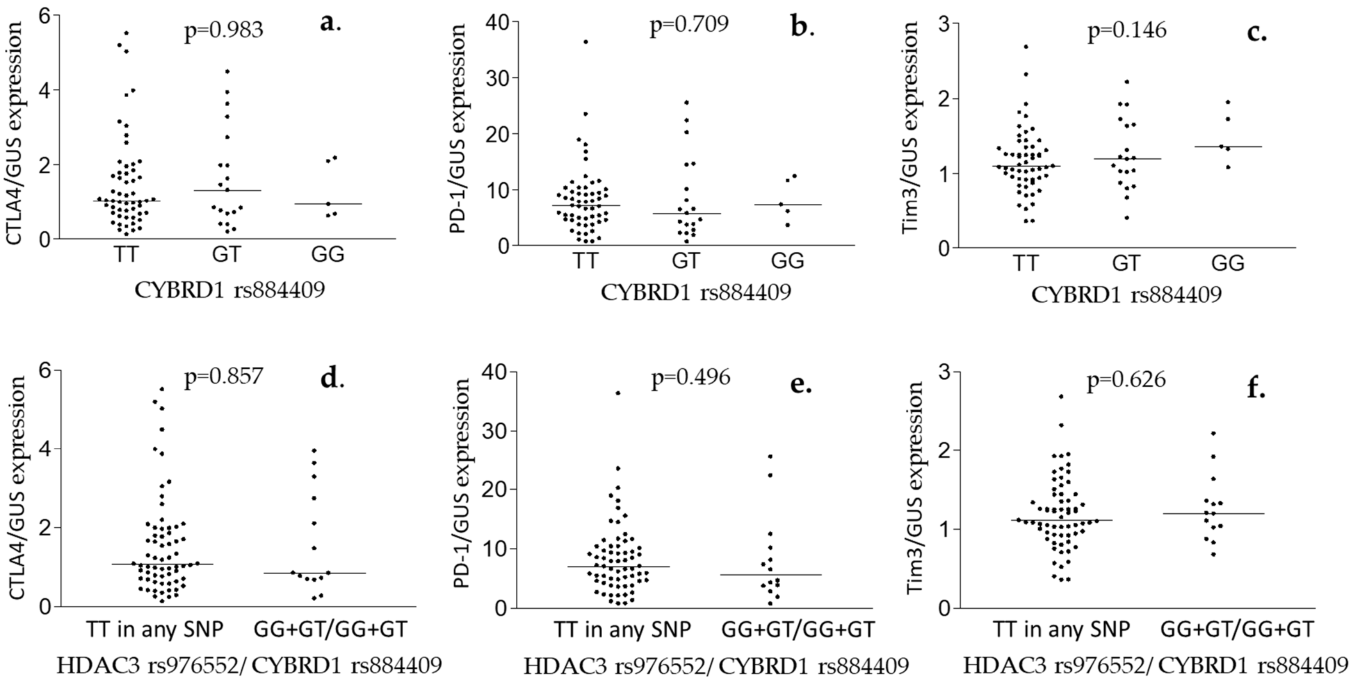

Figure S4. Hepatic expression of co-inhibitory receptors in relation to selected genotypes. Hepatic expression of *CTLA4* (**a, d**), *PD-1* (**b, e**), and *Tim3* (**c, f**) in samples from CHC patients with different *CYBRD1* rs884409 (**a**-**c**) and *HDAC3* rs976552/*CYBRD1* rs884409 (**d**-**f**) genotypes. Shown are the p values from a Kruskal-Wallis test (**a**-**c**) or Mann-Whitney U test (**d**-**f**).
